# Ghrelin Promotes Lipid Uptake into White Adipose Tissue via Endothelial Growth Hormone Secretagogue-Receptor in Mice

**DOI:** 10.3390/nu17010146

**Published:** 2024-12-31

**Authors:** Hidenori Urai, Tatsuhiko Azegami, Motoaki Komatsu, Rina Takahashi, Yoshiaki Kubota, Kazuhiro Hasegawa, Hirofumi Tokuyama, Shu Wakino, Kaori Hayashi, Takeshi Kanda, Hiroshi Itoh

**Affiliations:** 1 Division of Nephrology, Endocrinology and Metabolism, Department of Internal Medicine, Keio University School of Medicine, Shinjuku-ku, Tokyo 160-8582, Japan; h-urai@keio.jp (H.U.); mkomatsu0821@gmail.com (M.K.); 9rina22@gmail.com (R.T.); kaorihayashi@keio.jp (K.H.); hiito@keio.jp (H.I.); 2 Department of Anatomy, Keio University School of Medicine, Shinjuku-ku, Tokyo 160-8582, Japan; ykubo33@a3.keio.jp; 3 Division of Nephrology, Department of Internal Medicine, Tokushima University School of Medicine, Tokushima-shi 770-8503, Tokushima, Japan; kazuhiro@z2.keio.jp (K.H.); shuwakino@tokushima-u.ac.jp (S.W.); 4 Department of Internal Medicine, Tokyo Dental University Ichikawa General Hospital, Ichikawa-shi 272-8513, Chiba, Japan; hirobumitokuyama@aol.com; 5 Division of Nephrology, Department of Internal Medicine, Faculty of Medicine, Shimane University, Matsue 693-8501, Shimane, Japan; 6 The Center for Integrated Kidney Research and Advance (IKRA), Faculty of Medicine, Shimane University, Matsue 693-8501, Shimane, Japan

**Keywords:** endothelial cells, ghrelin, growth hormone secretagogue-receptor, obesity, triglyceride

## Abstract

**Background/Objectives**: Endothelial peroxisome proliferator-activated receptor gamma (PPARγ) regulates adipose tissue by facilitating lipid uptake into white adipocytes, but the role of endothelial lipid transport in systemic energy balance remains unclear. Ghrelin conveys nutritional information through the central nervous system and increases adiposity, while deficiency in its receptor, growth hormone secretagogue-receptor (GHSR), suppresses adiposity on a high-fat diet. This study aims to examine the effect of ghrelin/GHSR signaling in the endothelium on lipid metabolism. **Methods**: We compared the effects of ghrelin on adiposity and lipid uptake into adipocytes in wild-type and GHSR-null mice. Transgenic mice expressing GHSR selectively in endothelial cells were also generated and compared with global GHSR-null and wild-type mice. The impact of ghrelin on lipid uptake-related genes was assessed in cultured endothelial cells. **Results**: Ghrelin increased adiposity and triglyceride clearance in wild-type but not in GHSR-null mice. GHSR-null mice showed higher serum triglyceride after olive oil gavage and lower white adipose tissue (WAT) weight on a high-fat diet, suggesting impaired lipid uptake. Restoring GHSR expression in endothelial cells increased lipoprotein lipase activity, lipid uptake into WAT, and WAT weight. Ghrelin enhanced free fatty acid uptake and the expression of lipid uptake genes in cultured endothelial cells, whereas these effects were absent in GHSR-null mice-derived endothelial cells. Knockdown of PPARγ revealed that ghrelin/GHSR signaling in endothelial cells promoted lipid uptake via endothelial PPARγ. **Conclusions**: Endothelial GHSR is key for regulating lipid metabolism via PPARγ in response to ghrelin and for the role of endothelium in regulating white adipocyte metabolism. Targeting endothelial ghrelin signaling may be a promising therapeutic approach for managing excessive adiposity and associated metabolic disorders.

## 1. Introduction

Obesity is a global public health issue and a major cause of cardiovascular diseases [1]. Metabolic dysregulation in obesity, such as hyperglycemia, hypertension, dyslipidemia, and adipokine dysregulation, induces endothelial dysfunction [2]. Endothelial dysfunction, defined as decreased nitric oxide production and a reduced vasodilation response, is an important risk factor for atherosclerosis in individuals with obesity [3]. In addition to their role in atherosclerosis, endothelial cells (ECs) regulate adiposity by mediating lipid uptake into white adipose tissue (WAT). Furthermore, endothelial peroxisome proliferator-activated receptor gamma (PPARγ) activation facilitates free fatty acid (FFA) transport to adjacent adipocytes via the induction of lipid uptake genes, such as cluster of differentiation 36 (*Cd36*), fatty acid binding protein 4 (*Fabp4*), and glycosylphosphatidylinositol-anchored high-density lipoprotein-binding protein 1 (*Gpihbp1*), and increases lipid storage in WAT [4,5,6]. Although lipid uptake in WAT is likely regulated as part of systemic energy balance, the role of ECs in mediating this energy balance has not been fully characterized.

The gastric hormone ghrelin is stimulated by fasting and medium-chain FA ingestion and conveys nutritional information to the central nervous system (CNS) [7]. Ghrelin stimulates food intake and increases fat mass by binding to growth hormone secretagogue-receptor type 1a (GHSR) in the hypothalamus [7,8,9]. In addition to its orexigenic effect, the direct action of ghrelin on peripheral tissues, including WAT and the liver, increases lipid storage [10,11]: specifically, the ghrelin–GHSR axis augments lipid uptake via PPARγ induction in hepatocytes and adipocytes [11,12]. However, GHSR is also expressed in ECs and regulates endothelial function [13,14]; therefore, we hypothesized that ghrelin-induced adiposity is mediated by the action of endothelial GHSR, which increases lipid uptake from the circulation into white adipocytes.

Therefore, the aim of this this study is to clarify the role of endothelial GHSR in the mechanism of ghrelin-induced adiposity. To achieve this, we employed the following two approaches: (1) transgenic mice expressing GHSR selectively in ECs on a GHSR-null mouse background (GHSR-null/EC mice), using the Cre-LoxP system [15]; (2) the lipid uptake from circulation into white adipocytes in GHSR-null/EC mice was compared with that of global GHSR-null mice and wild-type (WT) mice; (3) based on the findings from the animal experiments, in vitro experiments were conducted to explore the signaling pathways involved in lipid uptake. The novelty of this study lies in elucidating the connection between two previously known phenomena: the involvement of endothelial cells in lipid uptake and the presence of GHSR in the vascular endothelium. Notably, this study reveals, for the first time, that ghrelin conveys nutrient status information to ECs in addition to the CNS, thus optimizing lipid storage in WAT by upregulating the expression of endothelial lipid uptake-related genes.

## 2. Materials and Methods

### 2.1. Animal Experiments

#### 2.1.1. Mice

GHSR-null mice, kindly provided by The University of Texas Southwestern Medical Center, Dallas, TX, USA, included a loxP-flanked transcriptional blocking cassette within the endogenous GHSR alleles [15,16]. We generated mice expressing GHSR selectively in ECs by crossing GHSR-null mice with mice expressing VE-Cadherin-Cre recombinase (Jackson Laboratory, Bar Harbor, ME, USA; stock number 006137) [17]. The transcriptional blocking cassette was removed from the recombinant null GHSR alleles in the presence of Cre recombinase, restoring GHSR expression. GHSR-null mice were on a C57BL/6 background and VE-Cadherin-Cre mice were backcrossed to C57BL/6 for at least five generations. VE-Cadherin-Cre mice were crossed with GHSR-null mice, and the offspring were bred with mice heterozygous for the GHSR-null allele to generate study animal genotypes: WT mice, with two WT GHSR alleles and no VE-Cad-Cre; GHSR-null mice, with two GHSR-null alleles and no VE-Cad-Cre; and GHSR-null/VE-Cad mice, with two GHSR-null alleles and one copy of VE-Cad-Cre. This removed the transcriptional blocking cassette in cells where Cre-recombinase was driven by the VE-Cad promoter (Supplementary Figure S1A). Genomic DNA from tail tips was extracted using a Direct PCR (tail) kit with proteinase K (Viagen Biotech Inc., Los Angeles, CA, USA). PCR was performed as previously reported with the following primers: M204, 5′-CGGTCTCCACCCTTCATTACTTTA-3′; M274, 5′-CAGATGTAGCTAAAAGGCCTATCACAAACT-3′; and M313, 5′-GATGCTTGGGGAAGAGAGAAGTGA-3′ (Supplementary Figure S1A) [18]. Mice carrying the VE-Cadherin-Cre transgene were identified using the following primers: 5′-GCGGTCTGGCAGTAAAAACTATC-3′ and 5′-GTGAAACAGCATTGCTGTCACTT-3′. The PCR product size was 102 bp.

The present studies in animals were reviewed and approved by the Institutional Animal Care and Use Committee of Keio University (approval number A2022-207) and was conducted in accordance with the ARRIVE guidelines [19]. Male mice were used in this study because their body weight increase in response to a high-fat diet (HFD) is significantly more pronounced than that of female mice, and the epididymal fat pad is easily isolated from male mice [20]. All mice were housed at a constant room temperature (22 ± 1 °C) and humidity (50–60%), under a 12 h light/dark cycle, with food and water provided ad libitum. Five animals were housed per cage. Age-matched male mice aged between 6 weeks and 6 months were included in this study. Before the study, all mice were fed a standard pellet diet (4.38% calories from fat; CE-2, CLEA Japan Inc., Tokyo, Japan). Obesity was induced using a HFD (Research Diets Inc., New Brunswick, NJ, USA; D12492), which contained 60% calories from fat, administered for 12 weeks starting at 6 weeks of age. The mice fed with a regular diet consumed a standard pellet diet (CE-2) throughout the study. For ghrelin treatment, acylated ghrelin peptide was synthesized (Eurofins Genomics, Tokyo, Japan), and 1.0 mg/kg acylated ghrelin was dissolved in normal saline and administered twice daily from Day 1 to Day 5 [21]. For control mice, normal saline was injected. Block randomization was performed considering the genotyping and weight of the animals. After the experiment, the mice were euthanized by cervical dislocation.

#### 2.1.2. Metabolic Testing

For the oral fat tolerance test, mice consuming standard chow were fasted overnight before olive oil gavage (10 mL/kg; Sigma-Aldrich, St. Louis, MO, USA); plasma triglyceride (TG) levels were then measured in tail vein blood. For ghrelin-treated mice, 1 h after ghrelin administration, mice were given olive oil. To bypass the effect of intestinal absorption, an intraperitoneal fat tolerance test was performed, and mice were injected peritoneally with 200 µL of intralipid 20% (vol/vol) fat emulsion (Otsuka Pharmaceutical, Tokyo, Japan), as described previously [22].

#### 2.1.3. Organ Distribution of Triolein-Derived 3H-Radioactivity

Oral fat tolerance tests were performed by gavage of 100 μL olive oil with [9,10-3H(N)]-triolein (370 kBq per mouse), as described previously [23]. For radioactivity measurement, organs and blood samples were solubilized in SOLVABLE (0.1 mL per 10 mg organ; PerkinElmer, Shelton, CT, USA); 200 μL of the solution was counted in scintillation fluid and TG-rich lipoprotein uptake was calculated as disintegrations per min (d.p.m.) per tissue [23].

#### 2.1.4. Serum Metabolites

Blood samples were collected from the tail vein 4 h after the start of the daily light cycle in ad libitum-fed mice [24]. Food was removed from cages at the start of the daily dark cycle, and fasting blood samples were collected 16 h later from the tail vein. Plasma FFAs, TG, and total cholesterol levels were determined using LabAssay NEFA, Triglyceride, and Cholesterol kits (Wako Chemicals, Osaka, Japan), respectively. Kits were used to assay plasma acylated ghrelin and unacylated ghrelin (Active Ghrelin ELISA Kit and Desacyl-Ghrelin ELISA Kit; SCETI K.K., Tokyo, Japan). Plasma lipoproteins were analyzed by high-performance liquid chromatography using molecular sieve columns (Skylight Biotech, Akita, Japan). Lipoprotein subclasses were defined based on lipoprotein particle size (diameter) [25].

#### 2.1.5. Chylomicron and VLDL Production

Four 16 h fasted mice were intraperitoneally injected with 1000 μg/g F-127 to inhibit lipoprotein lipase (LPL) activity [26]. Two minutes after the F-127 injection, mice received an oral gavage of olive oil to determine intestinal chylomicron (CM) production, as described previously [22]. For very low-density lipoprotein (VLDL) production, mice were injected with F-127 intraperitoneally without olive oil gavage. Blood was sampled before (0 h) and 2 and 4 h after injection. TG was measured enzymatically, as described above.

#### 2.1.6. Determination of LPL Activity in Post-Heparin Plasma

Two and a half hours after olive oil administration, mice were injected intraperitoneally with sodium heparin (200 U) [4]. After 30 min, venipuncture was performed, plasma was isolated and snap frozen, and lipoprotein lipase activity was assayed using a commercially available kit (LPL Activity Assay Kit; Cell Biolabs, Inc., San Diego, CA, USA).

#### 2.1.7. Histological Analysis

Paraffin-embedded WAT was analyzed by hematoxylin and eosin staining, before area measurement (Image Pro Plus 7.0 software; Media Cybernetics, Rockville, MD, USA) in more than five representative images and 300 cells per mouse. Tissue sections were coded to ensure that the evaluator was unaware of which sample belonged to which group.

### 2.2. In Vitro Experiments

#### 2.2.1. Mouse Endothelial Cell Culture

Microvascular ECs were isolated and cultured from the epididymal WAT of 2-week-old mice using Dynabead-conjugated antibodies against intercellular adhesion molecule 2 and CD31 (PharMingen, San Diego, CA, USA), as modified from previous methods [27]. In brief, epididymal adipose tissue was harvested from mice. Next, the tissue was incubated at 37 °C for 40 min in a digestive solution comprising 4% bovine serum albumin (BSA; Sigma-Aldrich) and 1.5 mg/mL collagenase type I (Worthington Biochemical Corp., Lakewood, NJ, USA). After filtration and centrifugation, pelleted cells were collected as the stromal vascular fraction. The pelleted cells were resuspended and incubated with CD31-coated Dynabeads at 4 °C with gentle agitation. A magnetic separator was used to recover bead-bound cells. The recovered cells were washed and suspended in culture medium (DMEM containing 20% fetal calf serum, supplemented with 5 mL 1% penicillin/streptomycin, 2.5 mL amphotericin B at 250 μg/mL, 10,000 units heparin [Wako Chemicals], and 50 mg endothelial cell growth factor [Biomedical Technologies, London, UK]) and plated in a collagen I-coated 10 cm^2^ tissue culture dish (Wako Chemicals). When the cells reached 70% to 80% confluence, they were detached using warm trypsin–ethylenediaminetetraacetic acid to generate a single-cell suspension. The cells were pelleted and resuspended in 2 mL PBS containing 0.1% BSA and sorted for a second time using intercellular adhesion molecule 2-coated beads. Bead-bound cells were washed and plated in complete culture medium and passaged further at a 1:2 ratio. Isolation of primary ECs was confirmed via CD31 staining with FACS analysis (Supplementary Figure S1B).

#### 2.2.2. Cell Culture and Experimental Protocol

Primary mouse ECs were seeded in 24-well collagen I-coated plates (Wako Chemicals) for examination of gene expression. Acyl-ghrelin at 10 nM (Peptide Institute Inc., Osaka, Japan) was added and incubated for 6 h. Cell lysates were then obtained. Human umbilical vein ECs (HUVECs) were treated with acyl-ghrelin (10 nM), with or without the mTOR inhibitor, rapamycin (20 nM) (LC Laboratories, Woburn, MA, USA), for 6 h.

#### 2.2.3. mRNA Isolation and Real-Time PCR

Total mRNA was isolated from ECs, treated with DNase I (Qiagen, Venlo, The Netherlands), and reverse transcribed (Takara Bio, Shiga, Japan). cDNA was subjected to SYBR Green real-time quantitative PCR (Real-Time PCR Detection System; Applied Biosystems, Waltham, MA, USA). Sequences for the sense and antisense primers were listed in Table 1.

The mRNA levels of these genes were quantified and expressed normalized to *Actb/ACTB* or *Rplp0* as an internal control. In addition, to perform the differential gene expression profiling in in ECs from WT and GHSR-null mice, Affymetrix ClariomTM S Arrays were used according to the manufacturer’s instructions (Affymetrix, St. Clara, CA, USA).

#### 2.2.4. siRNA Transfection

Human *PPARG* and control scramble siRNA were obtained as four oligonucleotide siGENOME SmartPools (Dharmacon Research Inc., Lafayette, CO, USA). HUVECs were transfected using Lipofectamine™ RNAiMAX reagent (Thermo Fisher Scientific, Waltham, MA, USA), according to the manufacturer’s instructions. Briefly, when cells reached approximately 70% confluence, they were starved in 1 mL serum-free endothelial basal medium for 2 h and incubated with Opti-MEM I medium containing 50 nM VDR siRNA mixed with Lipofectamine™ RNAiMAX transfection agent for 6 h. After transfection, cells were immediately plated in complete medium before performing assays (at 48 or 72 h).

#### 2.2.5. Transient Transfection and Luciferase Assay

The mouse endothelial cell line bEnd.3 was seeded into 96-well plates and transiently transfected with a PPAR response element (PPRE3)-TK-luciferase (generously provided by Dr. Jorge Plutzky, BWH) and a pNL1.1.PGK[Nluc/PGK] vector (Promega, Madison, WI, USA) using Lipofectamine 3000, according to the manufacturer’s protocol (Invitrogen, Waltham, MA, USA). Ninety-six hours after transfection, cells were preincubated in growth hormone-releasing peptide 6 (100 μM) (Abcam, Cambridge, UK) and Rapamycin (100 nM) for 1 h. Next, mouse acyl-ghrelin (100 nM) was added for 30 min and cell lysates were harvested. NanoLuc and firefly activities in cell lysates were measured using the Nano-Glo Dual-Luciferase Reporter Assay System (Promega). Dual luciferase signal was quantified using a Cytation 5 microplate reader (Agilent, Santa Clara, CA, USA). Firefly plasmid signal was normalized to NanoLuc signal.

#### 2.2.6. Measurement of Radioactively Labeled Long-Chain FA

Primary ECs from the WAT of WT and GHSR-null mice were starved in serum-reduced medium overnight. For determination of oleate uptake, cells were plated in 12-well plates and incubated with 1 uCi of [3H]oleate (GE Healthcare, Chicago, IL, USA) and 200 µmol/L oleate in DMEM containing 1% FFA–FBS albumin for 3 h at 37 °C. Cells were washed with PBS and lysed in 1 mL of 0.3 mol/L NaOH; the incorporated radioactivity was determined by scintillation counting. Protein content was quantitated using an assay kit (Wako Chemicals) [28].

#### 2.2.7. Transcytosis Assay

Primary ECs from the WAT of WT and GHSR-null mice were starved in serum-reduced medium overnight. For the determination of transcytosis, cells were plated in 24-well plates with Corning Costar Transwell inserts (6.5 mm diameter and 0.4 μm pore size), which were coated with collagen. The transcytosis assay was carried out as previously described [29]. Briefly, a long-chain FA analog, BODIPY-palmitic acid (PA) (BODIPY FL C16; Thermo Fisher Scientific) was used to measure the transcytosis of PA. BODIPY-PA (final concentration 40 μM) was put into the upper chamber of the noncompetitive insert, while BODIPY-PA (final concentration 40 μM) and a 10-fold-excess PA (final concentration 400 μM) were put into the upper chamber of the competitive insert. Next, 100 μL medium was collected from the lower chamber every hour and replaced with the same amount of medium to maintain the total well volume. The intensity of fluorescence was measured by a fluorescence spectrophotometer (Cytation 5). Excitation and emission wavelengths were 490 nm and 520 nm, respectively. Transcytosis of BODIPY-PA was calculated by subtracting the fluorescence reading of the competitive insert from that of the noncompetitive insert. The fluorescence intensity was normalized by protein concentrations of ECs using a protein assay kit (Wako Chemicals).

### 2.3. Statistical Analysis

Statistical analyses were performed using JMP 14.2 (SAS Institute, Cary, NC, USA). Data are expressed as mean ± standard error, with *p* < 0.05 being considered statistically significant. A one-way analysis of variance was used to determine significant differences among groups. For the overall analysis of variance, the Tukey–Kramer test for multiple comparisons was used to assess individual group differences.

## 3. Results

### 3.1. Ghrelin Increases Peripheral Lipid Clearance in Plasma and Stimulates Lipid Accumulation in WAT

**Figure 1:**
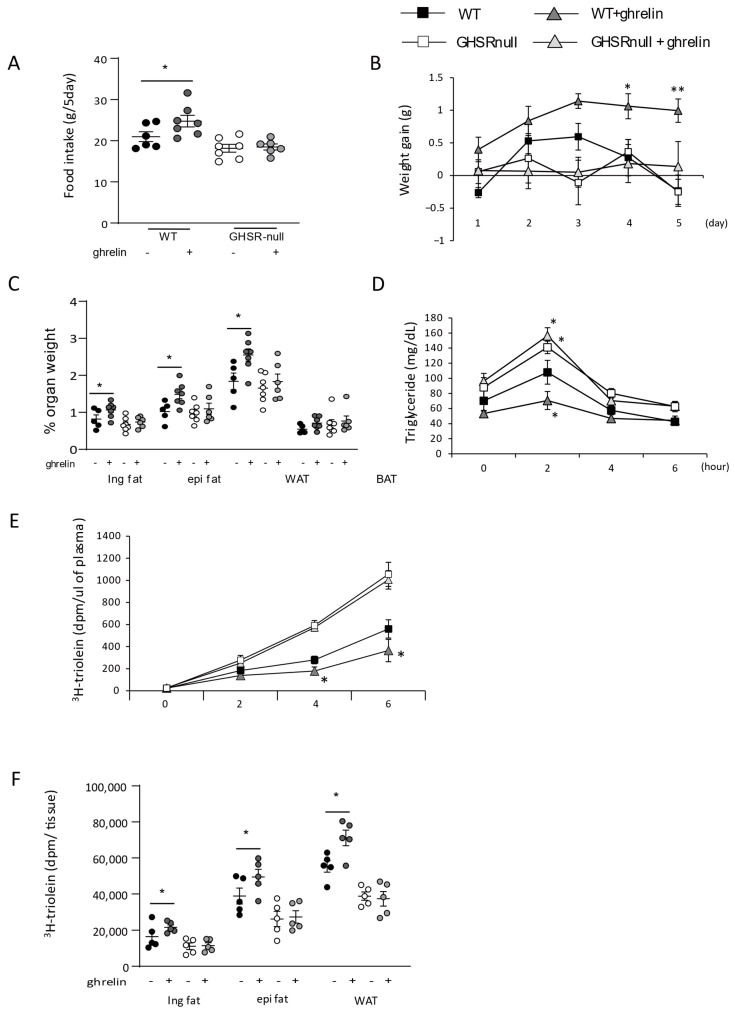
Ghrelin promotes adiposity in a growth hormone secretagogue-receptor (GHSR)-dependent manner. Food intake (**A**) and body weight gain (**B**) of wild-type (WT) mice and GHSR-null mice after acylated ghrelin treatment for 5 days. (**C**) Tissue weight as a percentage of total body weight for inguinal (ing), epididymal (epi), inguinal plus epididymal fat (white adipose tissue, WAT), and brown adipose tissue (BAT) after acylated ghrelin treatment. (**D**) Plasma triglyceride concentration after olive oil gavage. (**E**) Plasma 3H counts after oral gavage with 3H-triolein. (**F**) Tissue 3H counts derived from the same experiment. * *p* < 0.05, ** *p* < 0.01 versus WT mice without acylated ghrelin treatment (n = 5–7/group).

First, we investigated the effect of exogenous ghrelin on the regulation of adiposity and postprandial lipid levels in age-matched WT mice and GHSR-null mice. Ghrelin-treated WT mice had a significantly higher food intake and body weight than vehicle-treated WT mice, as previously reported (Figure 1A) [21,30]. This increase in body weight was associated with significantly higher WAT in ghrelin-treated WT mice (Figure 1B). However, when acylated ghrelin was administered to age-matched GHSR-null mice, ghrelin had no effect on food intake, weight gain (Figure 1A,B), or adiposity (Figure 1C). Lipid storage in WAT is determined by the balance of lipolysis and fat uptake in the fed state [31]: increased lipid uptake into WAT contributes to adiposity [4]. In humans, ghrelin treatment decreases postprandial lipid levels [32], implying that ghrelin increases TG clearance from the circulation. In line with this, we observed significantly lower plasma TG levels 2 h after olive oil gavage in ghrelin-treated WT mice than in vehicle-treated WT mice (Figure 1D). However, postprandial TG levels were significantly higher in GHSR-null mice (±ghrelin) than in vehicle-treated WT mice at this time point (Figure 1D). To eliminate the influence of endogenous TG, we performed an oral fat load test with olive oil mixed with 3H-triolein and found that plasma 3H-radioactivity was significantly lower in ghrelin-treated WT mice than GHSR-null mice (Figure 1E). Next, we examined the distribution of 3H-radioactivity in WAT after olive oil gavage and found that lipid uptake into WAT was significantly higher in ghrelin-treated WT mice than in vehicle-treated mice (Figure 1F). There was no effect of ghrelin on lipid uptake in the WAT of GHSR-null mice (Figure 1F), confirming that ghrelin treatment increased lipid uptake into WAT via GHSR.

**Figure 2:**
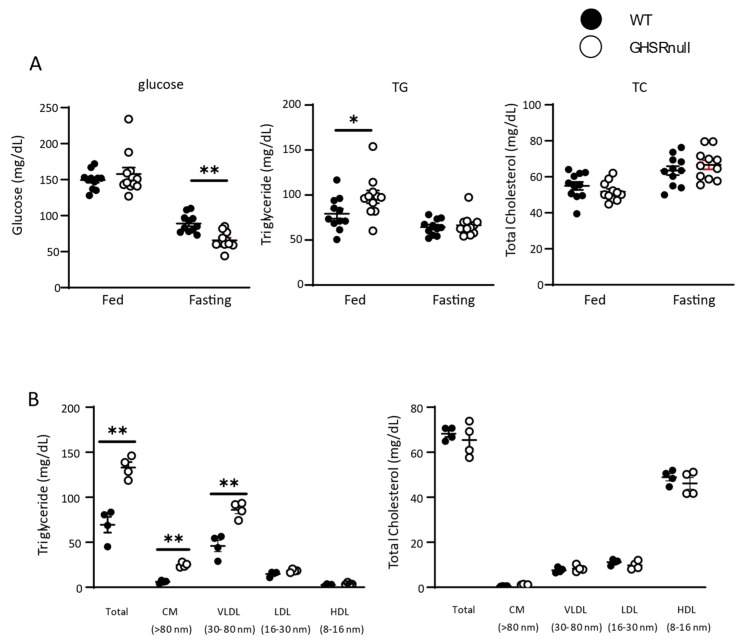
Growth hormone secretagogue-receptor (GHSR) increases postprandial lipid clearance. (**A**) Plasma glucose, triglyceride (TG), and total cholesterol (TC) levels were determined in fed and fasting wild-type (WT) and GHSR-null mice (n = 11/group). (**B**) Lipoprotein profiles 2 h after olive oil gavage (n = 4/group); CM, chylomicron; VLDL, very-low density lipoprotein; LDL, low-density lipoprotein; HDL, high-density lipoprotein. * *p* < 0.05, ** *p* < 0.01 versus WT mice.

We then examined the effect of GHSR on postprandial lipid levels. In the fasting state, plasma glucose was significantly lower in GHSR-null mice than in WT mice (Figure 2A), consistent with previous reports [24]. Meanwhile, in fed mice, plasma TG was significantly higher in GHSR-null mice than in WT mice (Figure 2A). Two hours after olive oil gavage, plasma TG levels in GHSR-null mice were significantly higher than those in WT mice (Figure 2B). High-performance liquid chromatography analysis showed that the increase in TG in GHSR-null mice was mainly due to increased CM and VLDL levels (Figure 2B); total cholesterol levels after olive oil gavage were not significantly different between mice (Figure 2B).

### 3.2. Restoring Endothelial GHSR Expression Augments Lipid Uptake

We previously reported that ECs regulate postprandial TG levels by increasing lipid uptake into WAT [4]. Meanwhile, GHSR is expressed in ECs, and the endothelium is a ghrelin target tissue [13,14]. Here, we first confirmed that ECs in WAT express GHSR by fluorescence microscopy (Supplementary Figure S1C). We further demonstrated that the expression of endothelial GHSR was higher in adipose tissue than in the liver and muscle (Supplementary Figure S1D).

**Figure 3:**
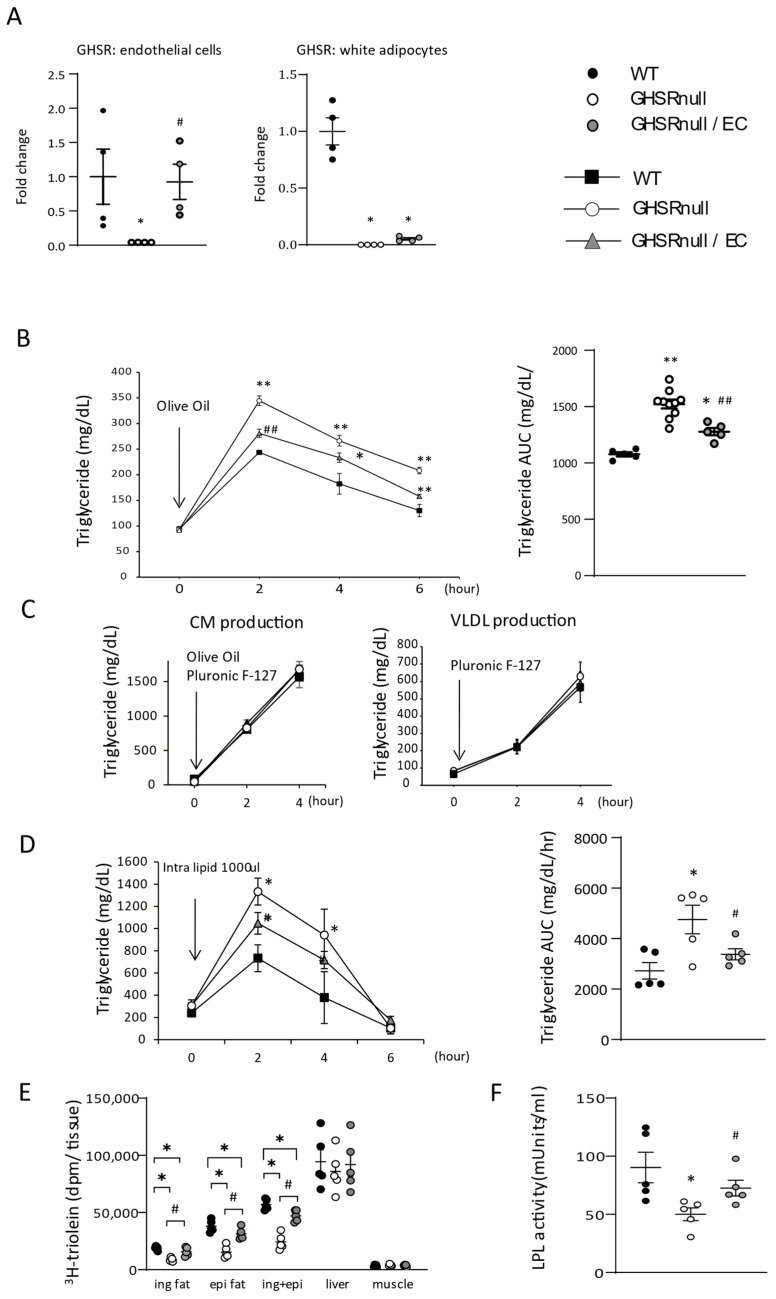
Endothelial growth hormone secretagogue-receptor (GHSR) increases triglyceride clearance by regulating lipoprotein lipase (LPL) activity. (**A**) Real-time quantitative PCR analysis of Ghsr expression in microvascular endothelial cells and white adipocytes isolated from wild-type (WT), GHSR-null, and GHSR-null/endothelial cell (EC) mice (n = 3–5/group). (**B**) Plasma triglyceride levels and areas under the curve (AUCs) after olive oil gavage in WT, GHSR-null, and GHSR-null/EC mice (n = 3–5/group). (**C**) Chylomicron (CM) production; plasma triglyceride concentrations after F-127 injection with olive oil gavage in overnight-fasted WT, GHSR-null, and GHSR-null/EC mice (n = 5/genotype). Very-low density lipoprotein (VLDL) production; plasma triglyceride concentrations after F-127 injection without olive oil gavage in overnight-fasted WT, GHSR-null, and GHSR-null/EC mice (n = 5/genotype). (**D**) Plasma triglyceride levels and AUCs after an intraperitoneal injection of lipid (n = 5/genotype). (**E**) Tissue 3H counts after oral gavage with 3H-triolein (n = 5/group); ing, inguinal; epi, epididymal. (**F**) Post-heparin LPL activity 3 h after olive oil gavage (n = 5/genotype). * *p* < 0.05, ** *p* < 0.01 versus WT mice. ^#^ *p* < 0.05, ^##^ *p* < 0.01 versus GHSR-null mice.

To elucidate the biological significance of endothelial GHSR in lipid metabolism by ghrelin in vivo, we generated transgenic mice expressing GHSR selectively in ECs (GHSR-null/EC mice) on a GHSR-null mouse background. GHSR-null/EC mice had significantly higher GHSR mRNA expression than GHSR-null mice in ECs but not adipocytes isolated from WAT (Figure 3A).

Next, we examined the role of endothelial GHSR in lipid metabolism. After olive oil administration, TG levels were significantly higher in GHSR-null mice than WT mice; however, TG levels in GHSR-null/EC mice (with expression of GHSR in ECs) were significantly lower than those in GHSR-null mice (Figure 3B). These results indicate that endothelial GHSR augments TG clearance from the circulation.

Postprandial plasma TG levels are primarily determined by the production and degradation of TG-rich lipoproteins, such as CM and VLDL. To determine the mechanisms underlying the lower lipid levels in GHSR-null/EC mice, we measured CM production in the intestine and VLDL production in the liver, and found no difference between WT, GHSR-null, and GHSR-null/EC mice (Figure 3C). In addition, to bypass intestinal absorption, we administered lipid intraperitoneally (intraperitoneal fat tolerance test) [22]. Despite this, GHSR-null mice continued to have a significantly higher plasma TG level than both GHSR-null/EC mice and WT mice (Figure 3D), suggesting increased clearance of TG-rich lipoprotein as the primary cause for lower lipid levels in GHSR-null/EC mice. Additionally, from the oral fat load test with 3H-triolein olive oil (Figure 3E), GHSR-null mice had significantly lower 3H-radioactivity in WAT than WT mice. Restoring the expression of GHSR in ECs resulted in a WAT 3H-radioactivity significantly higher than in GSHR-null mice and significantly lower than in WT mice. Therefore, the differences observed in plasma TG levels between the three groups following olive oil gavage were primarily attributable to differences in lipid uptake into WAT (Figure 3E). LPL is the key enzyme for the degradation of TG-rich lipoproteins, and GHSR-null mice had significantly lower plasma LPL activity than both WT and GHSR-null/EC mice (Figure 3F). These results suggest that endothelial GHSR increases TG clearance by increasing LPL activity.

### 3.3. Restoring Endothelial GHSR Expression Promotes Adiposity After a High-Fat Diet

**Figure 4:**
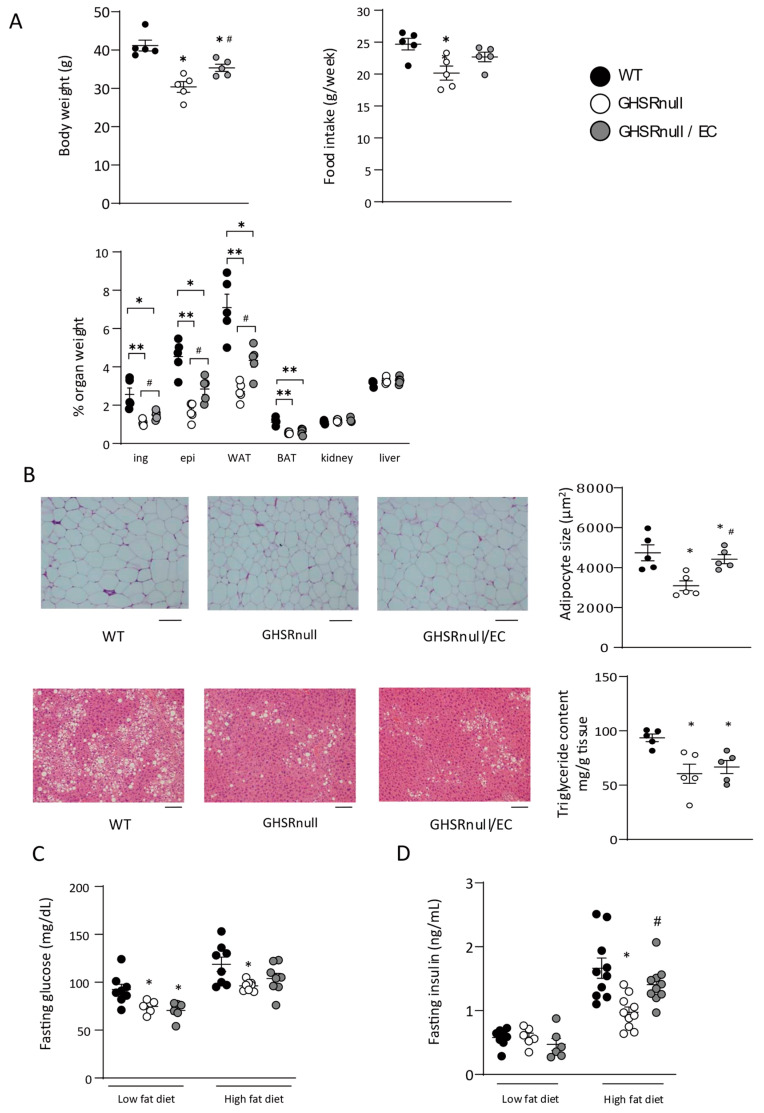

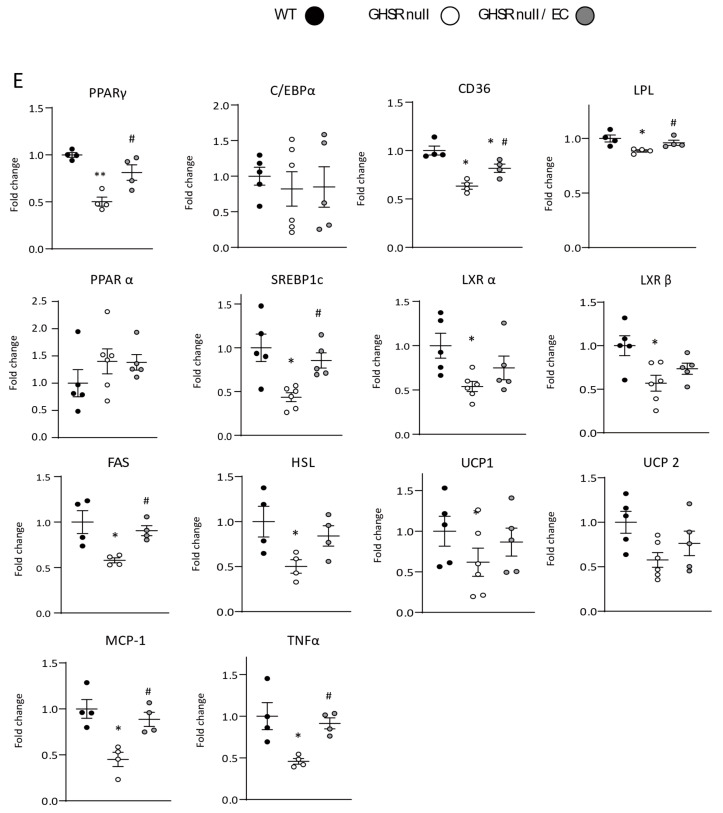
Endothelial growth hormone secretagogue-receptor (GHSR) promotes adiposity after high-fat diet intake. (**A**) Body weight, food intake, and percent organ weight of wild-type (WT), GHSR-null, and GHSR-null/endothelial cell (EC) mice fed a high-fat diet (HFD) for 12 weeks (n = 5–8 per group); ing, inguinal; epi, epididymal; WAT, white adipose tissue; BAT, brown adipose tissue. (**B**) Histology of epididymal adipose tissue and the liver of HFD-fed mice. Scale bars represent 100 µm. Mean adipocyte size in HFD-fed mice (n = 4–7/group). Liver triglyceride content in WT, GHSR-null, and GHSR-null/EC mice after the HFD (n = 5 per group). (**C**,**D**) Fasting glucose and insulin levels in WT, GHSR-null, and GHSR-null/EC mice on a low-fat diet and high-fat diet (n = 5–10/group). (**E**) mRNA expression of genes related to adipogenesis, lipid uptake, lipid synthesis, lipolysis, and lipid utilization in the WAT of HFD-fed WT, GHSR-null, and GHSR-null/EC mice. * *p* < 0.05, ** *p* < 0.01 versus WT mice. ^#^ *p* < 0.05 versus GHSR-null mice.

GHSR deletion throughout the body prevents HFD-induced adiposity and body weight gain [15], and therefore, we investigated the role of endothelial GHSR in the development of obesity after an HFD. We fed WT, GHSR-null, and GHSR-null/EC mice an HFD (60% calories derived from fat) for 12 weeks, beginning at 6 weeks of age. After feeding the HFD, body weight and fat percentage were significantly lower in GHSR-null mice than in WT mice and significantly higher in GHSR-null/EC mice than in GHSR-null mice (Figure 4A). Consistent with a previous report [15], HFD intake was significantly lower in GHSR-null mice (20.2 ± 1.1 g/week) than in WT mice (24.7 ± 0.9 g/week). However, restoring expression of GHSR in ECs resulted in a food intake similar to that of WT mice (22.7 ± 0.8 g/week) in GHSR-null/EC mice (Figure 4A). Inguinal and epididymal fat depots were significantly lower in GHSR-null mice than in WT mice (Figure 4A). Restoring the expression of GHSR in ECs resulted in a body weight and WAT weight gain significantly higher than in GHSR-null mice, but significantly lower than in WT mice (Figure 4A). Consistent with changes in adipose tissue weight, adipocyte size in epididymal WAT was 35% lower in HFD-fed GHSR-null mice than in WT mice, and restoring endothelial GHSR expression resulted in significantly larger adipocytes than in GHSR-null mice (Figure 4B). The ghrelin–GHSR axis promotes not only adiposity but also fatty liver [33]. Histological examination showed hepatic TG accumulation in HFD-fed WT mice, which was significantly higher than that in GHSR-null and GHSR-null/EC mice (Figure 4B).

Ghrelin regulates glucose homeostasis by multiple mechanisms such as reduction in insulin sensitivity, suppression of insulin secretion, stimulation of food intake and so on [34]. Fasting glucose levels on an LFD were significantly lower in GHSR-null mice compared to WT mice, and there was no difference in fasting glucose levels between GHSR-null mice and GHSR/EC-null mice, indicating that endothelial GHSR does not regulate fasting glucose level (Figure 4C). Fasting glucose levels on a HFD were significantly lower in GHSR-null mice compared to WT mice but not GHSR-null/EC mice (Figure 4C). Serum insulin levels were also lower in GHSR-null mice compared to WT mice, and reconstitution of GHSR in endothelial cells restored serum insulin levels compared with GHSR-null mice on a HFD (Figure 4D). These data suggested that glucose homeostasis improved by reduced adiposity.

HFD-fed GHSR-null mice had significantly lower expression of certain genes, such as *Lpl*, *Cd36*, *Srebp1c*, and *Fasn*, than WT mice in epidydimal adipose tissues (Figure 4E); restoring expression of GHSR in ECs resulted in significantly higher mRNA expression of these genes than in GHSR-null mice. Moreover, expression of the inflammatory markers Mcp1 and Tnf in the WAT of GHSR-null mice was significantly lower than in WT mice, while restoration of endothelial GHSR expression led to significantly higher expression of these genes than in GHSR-null mice. Therefore, these changes in WAT gene expression likely resulted from differences in endothelial GHSR expression.

### 3.4. Endothelial GHSR Regulates the Expression of Lipid Uptake Genes Induced by Ghrelin

**Figure 5:**
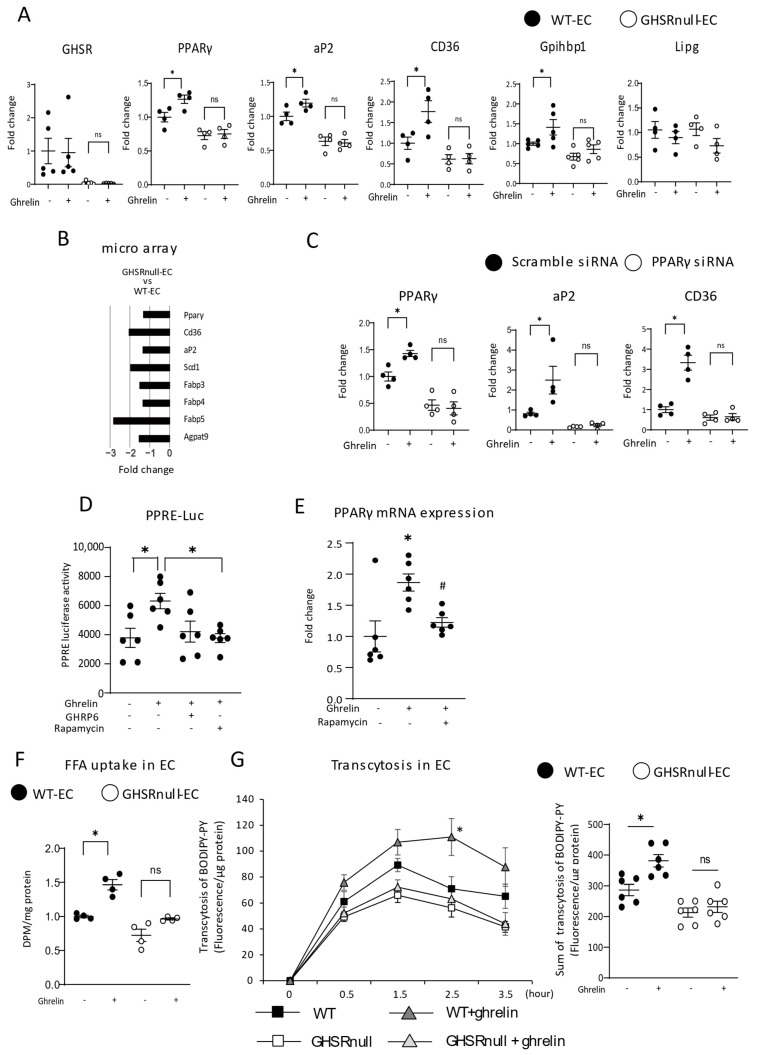
Endothelial growth hormone secretagogue-receptor (GHSR) regulates the expression of lipid uptake genes. (**A**) Expression of genes in primary microvascular endothelial cells (ECs) ± acylated ghrelin isolated from the white adipose tissue (WAT) of wild-type (WT) and GHSR-null mice. (n = 5–6/genotype) * *p* < 0.05 versus EC from WT mice without acylated ghrelin. (**B**) Microarray analysis for lipid metabolism genes in ECs from WT and GHSR-null mice. (**C**) PPARG mRNA levels in human umbilical vein endothelial cells (HUVECs) 48 h after siRNA transfection. Expression of genes in PPARG-knockdown HUVECs with or without acylated ghrelin treatment. (n = 4/group) * *p* < 0.05 versus HUVECs with scrambled siRNA without ghrelin. (**D**) The mouse endothelial cell line bEnd.3 was transfected with a PPAR response element (PPRE)–luciferase construct and exposed to ghrelin with or without growth hormone-releasing peptide 6 (GHRP-6) and rapamycin. (n = 6/group) * *p* < 0.05 versus bEnd.3 cells with ghrelin. (**E**) PPARG mRNA levels in HUVECs, with or without rapamycin. (n = 6/group) * *p* < 0.05 versus HUVECs without ghrelin. ^#^ *p* < 0.05. (**F**) Uptake of a radioactively labeled long-chain free fatty acid (FFA) by primary microvascular ECs isolated from the WAT of WT and GHSR-null mice without or with acylated ghrelin stimulation (n = 5–6/genotype). * *p* < 0.05 versus ECs from WT mice without acylated ghrelin. (**G**) Transcytosis of a long chain fatty acid analog, BODIPY-palmitic acid (PA) across primary ECs (n = 6). * *p* < 0.05 versus ECs from WT mice without acylated ghrelin. NS, not significant.

We examined the expression of genes associated with LPL activity and FFA uptake in ECs isolated from WAT. Ghrelin promotes lipogenesis via PPARγ induction [11]. In ECs isolated from WT mice, ghrelin significantly increased the mRNA expression of PPARγ (*Pparg*) and its target genes, such as *Cd36*, *Fabp4*, and *Gpihbp1* (Figure 5A). However, the effect of ghrelin on the expression of these genes was not evident in the ECs of GHSR-null mice. *Lipg* (endothelial lipase) is not a PPARγ target gene [4] and the mRNA expression level of *Lipg* was not different between groups (Figure 5A). We performed non-biased measurements of the expression levels of 22,900 annotated genes by microarray analysis in ECs from WT and GHSR-null mice (Figure 5B). Among lipid metabolism genes, eight, including *Cd36*, *Fabp4*, and *Pparg*, were downregulated in GHSR-null mice compared with WT mice. Next, we used *PPARG*-knockdown HUVECs to investigate whether the ghrelin–GHSR axis regulates lipid uptake in a PPARγ-dependent manner. We observed that ghrelin significantly increased *CD36* and *FABP4* mRNA expression in HUVECs; however, this effect was not evident in *PPARG*-knockdown HUVECs (Figure 5C).

Multiple intracellular signaling pathways are involved in the activation of GHSR [35]. Among them, mTOR signaling activated by ghrelin/GHSR is a key pathway for the regulation of energy metabolism [11,36] and activates PPARγ through increased phosphorylation of S6 [11]. To test whether the mTOR pathway mediated the observed results, the mouse endothelial cell line bEnd.3 was transfected with a PPRE-luciferase construct. Luciferase activity in these cells was significantly higher after ghrelin administration (Figure 5D), an effect abolished by pretreatment with the GHSR1a antagonist [D-Lys3]-growth hormone-releasing peptide-6 or the mTOR inhibitor rapamycin (Figure 5D). The transactivation activity of PPARγ increases its own expression [11]; we confirmed that Pparg expression was significantly increased by ghrelin and that this response was attenuated by rapamycin in endothelial cells (Figure 5E). Finally, we examined FFA uptake and transcytosis in ECs from WT and GHSR-null mice. Consistent with lower levels of lipid-regulating gene expression, ghrelin increased FFA uptake in WT ECs, but not in ECs lacking GHSR (Figure 5F). Moreover, the significantly higher transcytosis elicited by ghrelin in ECs from WT mice was not seen in ECs from GHSR-null mice (Figure 5G).

## 4. Discussion

Ghrelin is a key regulator of body weight and adiposity that functions by regulating food intake and energy expenditure primarily through the CNS. Although its receptor, GHSR, is expressed in peripheral tissues, such as the endothelium, the role of ghrelin signaling in ECs has not yet been fully elucidated. In the present study, we demonstrated that ghrelin promotes adiposity through endothelial GHSR. Endothelial ghrelin/GHSR signaling induced the expression of genes critical for lipid uptake by stimulating endothelial PPARγ. Our findings suggest that endothelial GHSR plays an important role in ghrelin-mediated increases in adiposity.

Ghrelin, originally referred to as the hunger hormone, induces a positive energy balance by signaling in the CNS to stimulate food intake and lower energy expenditure [37,38]. In addition, ghrelin functions as a dietary lipid sensor that signals a lipid-rich environment to the CNS, leading to lipid storage preparation in the body [39], which contributes to changes in lipid metabolism [40,41,42]. Unlike dietary long-chain FAs, medium-chain FAs can be efficiently absorbed into the circulation; therefore, medium-chain FAs can serve as substrates for acylated ghrelin and activate the ghrelin system [43]. In this study, we found that endothelial ghrelin/GHSR signaling promoted FFA uptake into WAT and adiposity in response to acylated ghrelin administration. Therefore, it seems that ghrelin conveys nutrient status information to ECs in addition to the CNS, thus optimizing lipid storage in WAT via upregulation of endothelial lipid uptake-related gene expression.

Ghrelin increases adiposity by acting centrally and/or peripherally; it exists as two major endogenous forms, acylated at serine 3 (acylated ghrelin) and not acylated at serine 3 (des-acylated ghrelin). Acylated ghrelin activates GHSR and increases food intake [9], whereas des-acylated ghrelin increases food intake independent of GHSR [44]. In this study, we administered acylated ghrelin to mice (Figure 1). Hence, the phenotypes observed in this study are likely dependent on the acylated ghrelin/GHSR pathway.

Neuronal GHSR decreases energy expenditure by suppressing thermogenesis and physical activity without affecting food intake [45]. Here, we observed that endothelial GHSR partially restored ghrelin-induced changes in adiposity (Figure 4A). These results suggest that peripheral ghrelin signaling, particularly in ECs, contributes to the effect of ghrelin. Previously, we, and another group, demonstrated that increased lipid uptake through ECs contributes to adipose fat accumulation in response to an HFD [4,6]. Therefore, increases in lipid uptake through endothelial GHSR lead to fat accumulation in HFD-fed mice. The expansion of WAT and increased adipogenic gene expression are regulated by FFA uptake in white adipocytes. Ghrelin controls adipocyte metabolism by acting directly on adipocytes [46] and/or the CNS [47]. In this study, adipocytes in HFD-fed GHSR-null/EC mice were significantly larger than those in HFD-fed GHSR-null mice. Changes in adipogenic gene expression in GHSR-null/EC mice were consistent with these changes in adipocyte size, although both GHSR-null mice and GHSR-null/EC mice lacked GHSR in white adipocytes (Figure 3A). Specifically, certain FAs regulate adipogenic genes in white adipocytes by binding directly to PPARγ [48]. We observed that endothelial GHSR facilitates FA uptake and transcytosis (Figure 5F,G), suggesting increased FFA delivery to WAT. Moreover, the expression of lipid uptake genes (Lpl and Cd36) as well as a lipid synthesis gene (Fasn), was decreased in GHSR-null mice (Figure 4E). Therefore, we speculate that changes in gene expression in WAT might be attributed to increased availability of FFA delivered via the action of endothelial GHSR.

ECs are known as gatekeepers for the cellular uptake of circulating nutrients. LPL is a key enzyme in the regulation of FFA flux into WAT. GPIHBP1 transports LPL from white adipocytes to the surface of ECs [49], where LPL hydrolyzes TG-rich lipoproteins, such as CM and VLDL, and liberates FFAs [50]. Restoring endothelial GHSR expression in GHSR-deficient mice partially restored plasma LPL activity and lipid uptake in WAT (Figure 3). Increased LPL mRNA expression (*Lpl*) in WAT (Figure 4E) and endothelial Gpihbp1 expression [49] (Figure 5A) likely contribute to increased LPL activity in GHSR-null/EC mice. FFAs liberated by LPL hydrolysis are transported into white adipocytes through endothelial FA transporters, such as CD36 and FABP4 [50]. PPARγ functions as a master regulator of adipocyte differentiation and lipid metabolism, and endothelial PPARγ regulates lipid uptake genes, such as *Cd36*, *Fabp4*, and *Gpihbp1* [4]. We found that acylated ghrelin/GHSR signaling induces lipid uptake genes in a PPARγ-dependent manner through the mTOR pathway (Figure 5D).

Ghrelin exerts beneficial effects on endothelial function, such as the production of nitric oxide [51] and the inhibition of proinflammatory cytokines [14], thereby alleviating endothelial dysfunction in obesity [52]. However, the protective effect of ghrelin against atherosclerosis is unclear [53]. Here, we demonstrated that restoring the expression of GHSR in ECs increases adiposity, which might in turn lead to endothelial dysfunction. Thus, the beneficial effects of ghrelin on the cardiovascular system may be negated by its effect on adiposity [44].

The ghrelin–GHSR axis increases not only body weight and adiposity, but also fatty liver [33]. In this study, we found that reconstitution of endothelial GHSR in GHSR-null mice increased body weight and adiposity in WAT; however, it did not affect the liver. Reconstitution of GHSR in ECs tended to increase food intake (22.7 ± 0.8 g/week) compared with GHSR-null mice (Figure 4A). Although the difference in food intake (2.5 g/week) was not significant, it may have contributed to the increased weight gain observed in GHSR-null/EC mice. Endothelial GHSR also increased lipid uptake by increasing LPL activity (Figure 3F), which correlates with food intake [54]. For instance, mice deficient in Apolipoprotein C3, a strong LPL inhibitor, demonstrate increased LPL activity, fat accumulation, and food intake [55]. Therefore, increased LPL activity via reconstitution of endothelial GHSR may explain the changes in body weight and food intake observed in GHSR-null/EC mice. Moreover, GHSR in hepatocytes promotes TG accumulation in the liver [11]. Here, we observed that total TG content was decreased in the liver of GHSR-null mice (Figure 4B), while accumulation of hepatic TG content was similar between GHSR-null mice and GHSR-null/EC mice. GHSR deficiency in hepatocytes did not differ between the GHSR-null mice and GHSR-null/EC mice. Moreover, lipid uptake by the liver is passive and ECs do not have an integral role in lipid clearance by the liver as the endothelium is fenestrated and remnant lipoproteins are transported freely. Indeed, lipid uptake in the liver was similar between the three groups (Figure 3E), which may explain the observed liver phenotype of the three study groups.

Previous reports have shown that ghrelin induces tissue-specific changes in the expression of mitochondrial and lipid metabolism-related genes, promoting triglyceride deposition in the liver while inhibiting it in skeletal muscle [56]. Additionally, peripheral ghrelin has been reported to increase lipid accumulation in a depot-specific manner in WAT through GHSR-mediated lipolysis [46]. In contrast to these existing findings, our study focuses not on peripheral organs or peripheral adipose tissue, but rather on the lipid uptake action mediated by ghrelin/GHSR in ECs, clarifying the mechanism of fat accumulation. This approach represents a highly novel contribution to the field.

This study suggests that ghrelin/GHSR signaling promotes lipid uptake and WAT accumulation via endothelial cells, making this pathway a promising therapeutic target for obesity and dyslipidemia. Inhibition of GHSR could suppress lipid uptake in endothelial cells, potentially reducing fat accumulation and improving metabolic disorders associated with obesity. However, since there is currently no medication to specifically inhibit GHSR in endothelial cells, the impact of systemic GHSR inhibition on the beneficial cardiovascular effects of ghrelin remains unclear, necessitating further investigation. Similarly, this study is based on animal models, and the effects and safety of GHSR inhibition in humans have not been sufficiently explored. Moreover, the long-term health impacts of complete GHSR inhibition, particularly its effects on hormone balance and metabolic function, are unknown and need to be considered. Future research should clarify the mechanisms underlying lipid metabolism improvement through GHSR inhibition and establish the safety and efficacy for clinical application in humans.

## 5. Conclusions

We demonstrated that endothelial GHSR plays a critical role in promoting adiposity and weight gain by regulating specific endothelial PPARγ target genes. Our results suggest that ghrelin not only acts as a CNS-mediated signal for energy storage but also functions as an important peripheral regulator of lipid metabolism via endothelial signaling. Specifically, endothelial GHSR activation enhances lipid uptake into WAT, contributing to an increase in adiposity. These findings highlight a novel, non-CNS pathway through which ghrelin exerts its metabolic effects, broadening our understanding of its role in systemic energy balance. Furthermore, hormonal changes affect chronic conditions such as lifestyle-related diseases; therefore, targeting endothelial ghrelin signaling may be a promising and specific therapeutic strategy for managing excessive adiposity and associated metabolic disorders.

## Figures and Tables

**Table 1:** The sequence of sense and antisense primers.

**For Mouse Experiments**	
*Pparg*	5′-CAAGAATACCAAAGTGCGATCAA-3′
	5′-GAGCAGGGTCTTTTCAGAATAATAAG-3′
*Cebpa*	5′-CGCAAGAGCCGAGATAAAGC-3′
	5′-CACGGCTCAGCTGTTCCA-3′
*Cd36*	5′-GGCCAAGCTATTGCGACAT-3′
	5′-CAGATCCGAACACAGCGTAGA-3′
*Lpl*	5′-GTGGCCGAGAGCGAGAAC-3′
	5′-AAGAAGGAGTAGGTTTTATTTGTGGAA-3′
*Srebp1c*	5′-GGAGCCATGGATTGCACATT-3′
	5′-CCTGTCTCACCCCCAGCATA-3′
*Nrlh3*	5′-CTGCGATTGAGGTGATGCTC-3′
	5′-CGGTCTGCAGAGAAGATGC-3′
*Nrlh2*	5′-TGACGTGGCGGAGGTACTG-3′
	5′-CCCCACAAGTTCTCTGGACACT-3′
*Fasn*	5′-ACCACCAGAGACCGTTATGC3′
	5′-TGGGTTCTAGCCAGCAGAGT-3′
*Lipe*	5′-GCTGGGCTGTCAAGCACTGT-3′
	5′-GTAACTGGGTAGGCTGCCA-3′
*Ppara*	5′- AGGAAGCCGTTCTGTGACAT-3′
	5′-AATCCCCTCCTGCAACTTCT-3′
*Ccl2*	5′-TGATCCTCTTGTAGCTCTCCAGC-3′
	5′-TCAGCCAGATGCAGTTAACGC-3′
*Tnf*	5′-CCACCACGCTCTTCTGTCTA-3′
	5′-AGGGTCTGGGCCATAGAACT-3′
*Fabp4*	5′-TGGAAGCTTGTCTCCAGTGA-3′
	5′-AATCCCCATTTACGCTGATG-3′
*Gpihbp1*	5′-TCTTGCTACTAAGTGGACAGCCAG-3′
	5′-TGCTTCCAGGGATCATGTTGGTCT-3′
*Ghsr*	5′-GGGACCAGAACCACAAACAG-3′
	5′-GGAAAACAGATATCTTCCCACG-3′
*Actb*	5′-CAGCCTTCCTTCTTGGGTATGG-3′
	5′-CTGTGTTGGCATAGAGGTCTTTACG-3′
*Lipg*	5′-ATGCGAAACACGGTTTTCCTG-3′
	5′-GTAGCTGGTACTCCAGTGGG-3′
*Rplp0*	5′-GGCCCTGCACTCTCGCTTTC-3′
	5′-TGCCAGGACGCGCTTGT-3′
**For human umbilical vein endothelial cell experiments**	
*PPARG*	5′-AGCCTCATGAAGAGCCTTCCA-3′
	5′-TCCGGAAGAAACCCTTGCA3′
*FABP4*	5′-CAACGTCCCTTGGCTTATGCT-3′
	5′-TGTGCAGAAATGGGATGGAAA-3′
*CD36*	5′-CAGAGGCTGACAACTTCACAG-3′
	5′-AGGGTACGGAACCAAACTCAA-3′
*ACTB*	5′-AGCACTGTGTTGGCGTACAG-3′
	5′-GGACTTCGAGCAAGAGATGG-3′

## Data Availability

All data generated and/or analyzed during this study are included in this published article and its Supplementary Materials.

## Supplementary Materials

The following supporting information can be downloaded at: https://www.mdpi.com/article/10.3390/nu17010146/s1, Figure S1: Characteristics of the endothelial growth hormone secretagogue receptor (GHSR).
