# Supplementary figures and images for: Ghrelin Promotes Lipid Uptake into White Adipose Tissue via Endothelial Growth Hormone Secretagogue-Receptor in Mice
Source: Nutrients. 2024 Dec 31;17(1):146. doi: 10.3390/nu17010146 (PMC11722803; doi:10.3390/nu17010146)

A

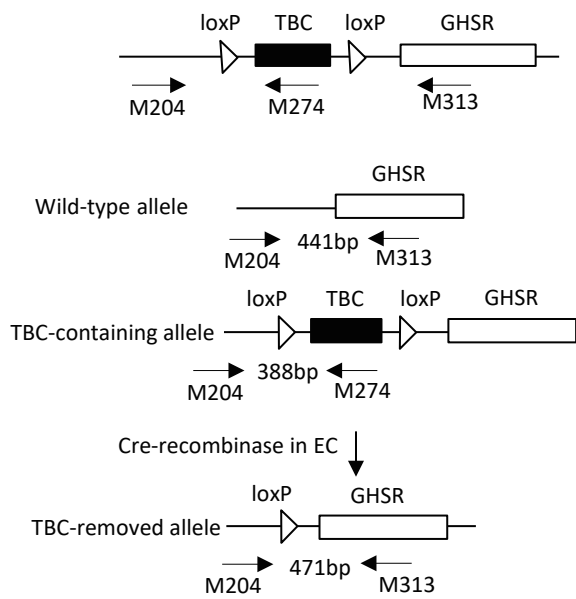

B

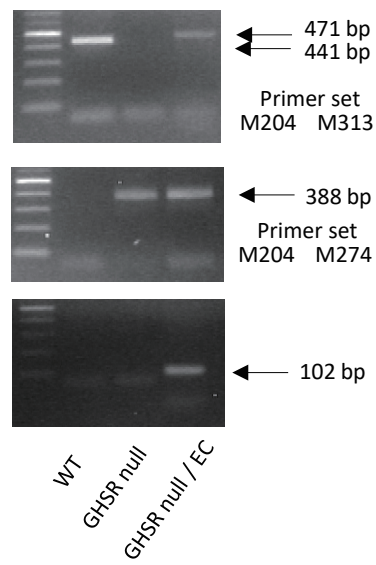

CD31 positive cells

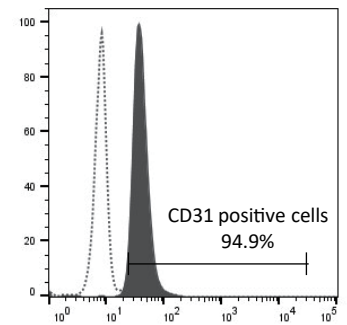

C

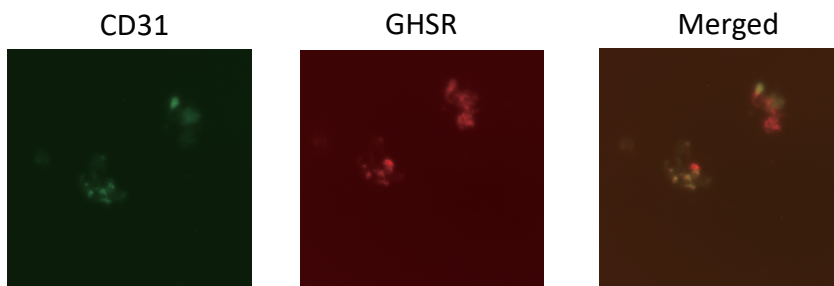

D

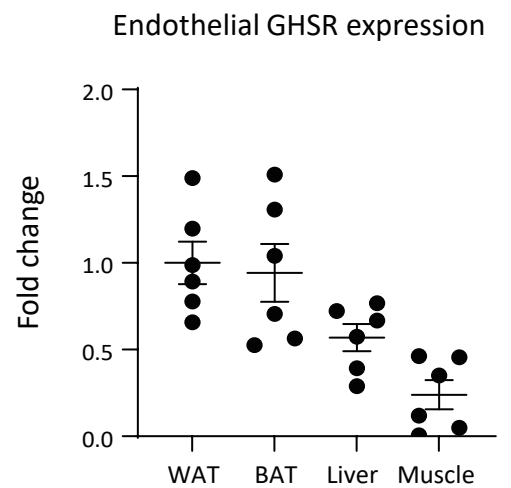

Supplement: Supplementary file 1 [file nutrients-17-00146-s001.zip › nutrients-3399752-supplementary.pdf]
